# 3-(4-Methoxy­phen­yl)-1*H*-isochromen-1-one

**DOI:** 10.1107/S1600536808042074

**Published:** 2008-12-17

**Authors:** T. Maiyalagan, Venkatesha R. Hathwar, P. Manivel, N. Burcu Arslan, F. Nawaz Khan

**Affiliations:** a School of Science and Humanities, VIT University, Vellore 632 014, Tamil Nadu, India; b Solid State and Structural Chemistry Unit, Indian Institute of Science, Bangalore 560 012, Karnataka, India; c Ondokuz Mayıs University, Arts and Sciences Faculty, Department of Physics, 55139-Samsun, Turkey

## Abstract

The asymmetric unit of the title compound, C_16_H_12_O_3_, contains two crystallographically independent mol­ecules. The isochromene ring system is planar (maximum deviation 0.024 Å) and is oriented at dihedral angles of 2.63 (3) and 0.79 (3)° with respect to the methoxy­benzene rings in the two independent mol­ecules.

## Related literature

For general background, see: Barry (1964 ▶); Hill (1986 ▶); Canendo *et al.* (1997 ▶); Whyte *et al.* (1996 ▶). For related structures, see: Abid *et al.* (2006 ▶, 2008 ▶); Hathwar *et al.* (2007 ▶).
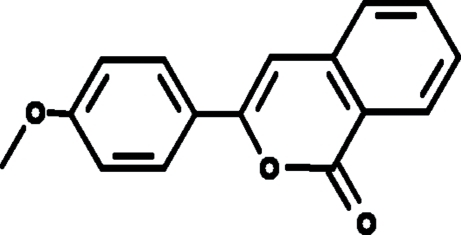


## Experimental

### 

#### Crystal data

C_16_H_12_O_3_
*M* *_r_* = 252.26
Monoclinic, 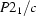

*a* = 15.5949 (15) Å
*b* = 11.8464 (11) Å
*c* = 15.1824 (14) Å
β = 117.838 (2)°
*V* = 2480.2 (4) Å^3^
*Z* = 8
Mo *K*α radiation
μ = 0.09 mm^−1^
*T* = 290 (2) K
0.28 × 0.14 × 0.08 mm

#### Data collection

Bruker SMART CCD area-detector diffractometer
Absorption correction: multi-scan (*SADABS*; Sheldrick, 1996 ▶) *T*_min_ = 0.974, *T*_max_ = 0.984
18262 measured reflections
4616 independent reflections
2795 reflections with *I* > 2σ(*I*)
*R*_int_ = 0.038

#### Refinement

*R*[*F* ^2^ > 2σ(*F* ^2^)] = 0.045
*wR*(*F* ^2^) = 0.109
*S* = 1.00
4616 reflections
345 parameters
All H-atom parameters refined
Δρ_max_ = 0.15 e Å^−3^
Δρ_min_ = −0.15 e Å^−3^

### 

Data collection: *SMART* (Bruker, 2004 ▶); cell refinement: *SAINT* (Bruker, 2004 ▶); data reduction: *SAINT*; program(s) used to solve structure: *SHELXS97* (Sheldrick, 2008 ▶); program(s) used to refine structure: *SHELXL97* (Sheldrick, 2008 ▶); molecular graphics: *ORTEP-3* (Farrugia, 1997 ▶) and *CAMERON* (Watkin *et al.*, 1993 ▶); software used to prepare material for publication: *PLATON* (Spek, 2003 ▶).

## Supplementary Material

Crystal structure: contains datablocks global, I. DOI: 10.1107/S1600536808042074/nc2128sup1.cif

Structure factors: contains datablocks I. DOI: 10.1107/S1600536808042074/nc2128Isup2.hkl

Additional supplementary materials:  crystallographic information; 3D view; checkCIF report

## Figures and Tables

**Fig. 1.**
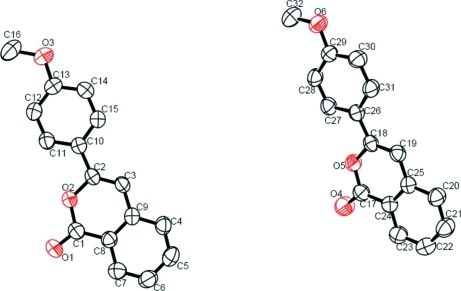
: Crystal structure of the title complex, showing 50% probability displacement ellipsoids and the atom-numbering scheme.

## supplementary crystallographic information

### Comment

Isochromenones are structurally related to the chromenones, (Hill, 1986). They have a wide range of biological activities (Hill, 1986; Canendo *et al.*, 1997; Whyte *et al.*, 1996). Isocoumarins (Barry, 1964) are also useful intermediates in the synthesis of a variety of important compounds including some carbocyclic and heterocyclic compounds. In view of their natural occurrence, biological activities and utility as synthetic intermediates, we have synthesized the title compound, and reported herein its crystal structure.

The asymmetric unit of the title compound contains two crystallographically independent molecules of similar geometry. The dihedral angels between the isochromene ring system and the methoxybenzene rings amount to 2.63 (3) and 0.79 (3) ° in the two crystallographically independent molecules

### Experimental

Homophthalic acid (1.3 g, 7.2 mmol) was added to *p*-methoxybenzoyl chloride (24.8 mmol) and was refluxed for 4 h at 473 K with stirring. The reaction mixture was extracted with ethyl acetate (3 times 100 ml), and an aqueous solution of sodium carbonate (5%, 200 ml) was added to remove the unreacted homophthalic acid. The organic layer was separated, concentrated and chromatographed on silica gel using petroleum ether (313–353 K fractions) as eluent to afford the title compound. Single crystals suitable for X-ray analysis were obtained by slow evaporation of an ethyl acetate solution.

### Refinement

All H atoms were positioned with idealized geometry and were refined using a riding model with C-H = 0.96 Å for CH_3_ and 0.93 Å for aromatic H atoms. The displacement parameters of the H atoms were constrained as *U*_iso_(H) = 1.2*U*_eq_ (1.5*U*_eq_ for methyl) of the carrier atom.

### Crystal data

**Table:**

C_16_H_12_O_3_	*F*(000) = 1056
*M**_r_* = 252.26	*D*_x_ = 1.351 Mg m^−^^3^
Monoclinic, *P*2_1_/*c*	Mo *K*α radiation, λ = 0.71073 Å
Hall symbol: -P 2ybc	Cell parameters from 8668 reflections
*a* = 15.5949 (15) Å	θ = 1.5–25.5°
*b* = 11.8464 (11) Å	µ = 0.09 mm^−^^1^
*c* = 15.1824 (14) Å	*T* = 290 K
β = 117.838 (2)°	Block, colourless
*V* = 2480.2 (4) Å^3^	0.28 × 0.14 × 0.08 mm
*Z* = 8	

### Data collection

**Table:**

Bruker SMART CCD area-detector diffractometer	4616 independent reflections
Radiation source: fine-focus sealed tube	2795 reflections with *I* > 2σ(*I*)
graphite	*R*_int_ = 0.038
φ and ω scans	θ_max_ = 25.5°, θ_min_ = 1.5°
Absorption correction: multi-scan (*SADABS*; Sheldrick, 1996)	*h* = −18→18
*T*_min_ = 0.974, *T*_max_ = 0.984	*k* = −13→14
18262 measured reflections	*l* = −18→18

### Refinement

**Table:**

Refinement on *F*^2^	Primary atom site location: structure-invariant direct methods
Least-squares matrix: full	Secondary atom site location: difference Fourier map
*R*[*F*^2^ > 2σ(*F*^2^)] = 0.045	Hydrogen site location: inferred from neighbouring sites
*wR*(*F*^2^) = 0.109	All H-atom parameters refined
*S* = 1.00	*w* = 1/[σ^2^(*F*_o_^2^) + (0.0567*P*)^2^] where *P* = (*F*_o_^2^ + 2*F*_c_^2^)/3
4616 reflections	(Δ/σ)_max_ = 0.001
345 parameters	Δρ_max_ = 0.14 e Å^−^^3^
0 restraints	Δρ_min_ = −0.15 e Å^−^^3^

### Special details

**Table:**

Geometry. All e.s.d.'s (except the e.s.d. in the dihedral angle between two l.s. planes) are estimated using the full covariance matrix. The cell e.s.d.'s are taken into account individually in the estimation of e.s.d.'s in distances, angles and torsion angles; correlations between e.s.d.'s in cell parameters are only used when they are defined by crystal symmetry. An approximate (isotropic) treatment of cell e.s.d.'s is used for estimating e.s.d.'s involving l.s. planes.

### Fractional atomic coordinates and isotropic or equivalent isotropic displacement parameters (Å^2^)

**Table:**

	*x*	*y*	*z*	*U*_iso_*/*U*_eq_	
O1	0.46573 (12)	0.71997 (11)	1.12417 (10)	0.0894 (5)	
O2	0.41915 (8)	0.88630 (10)	1.05390 (8)	0.0602 (3)	
O3	0.35416 (9)	1.40912 (10)	1.06979 (10)	0.0762 (4)	
O4	0.03964 (12)	0.71696 (11)	0.15948 (11)	0.0912 (5)	
O5	0.08196 (8)	0.88474 (10)	0.13156 (8)	0.0595 (3)	
O6	0.13583 (9)	1.40934 (10)	0.20493 (9)	0.0667 (4)	
C1	0.43111 (14)	0.77173 (16)	1.04693 (14)	0.0606 (5)	
C2	0.38013 (11)	0.95680 (14)	0.97170 (12)	0.0470 (4)	
C3	0.35328 (11)	0.91498 (14)	0.88124 (12)	0.0496 (4)	
H3	0.3276	0.9634	0.8267	0.060*	
C4	0.33679 (13)	0.75059 (16)	0.77199 (13)	0.0600 (5)	
H4	0.3121	0.7967	0.7159	0.072*	
C5	0.34738 (13)	0.63695 (17)	0.76232 (14)	0.0667 (5)	
H5	0.3305	0.6069	0.6998	0.080*	
C6	0.38271 (13)	0.56691 (17)	0.84417 (15)	0.0690 (5)	
H6	0.3885	0.4899	0.8364	0.083*	
C7	0.40934 (13)	0.61035 (16)	0.93675 (14)	0.0646 (5)	
H7	0.4332	0.5630	0.9920	0.077*	
C8	0.40056 (12)	0.72591 (15)	0.94798 (12)	0.0503 (4)	
C9	0.36294 (11)	0.79755 (14)	0.86563 (12)	0.0475 (4)	
C10	0.37489 (11)	1.07382 (14)	1.00008 (12)	0.0467 (4)	
C11	0.40524 (12)	1.10565 (15)	1.09812 (12)	0.0551 (5)	
H11	0.4297	1.0508	1.1476	0.066*	
C12	0.40041 (13)	1.21545 (16)	1.12477 (13)	0.0581 (5)	
H12	0.4221	1.2340	1.1913	0.070*	
C13	0.36364 (13)	1.29749 (15)	1.05313 (14)	0.0552 (5)	
C14	0.33210 (13)	1.26874 (16)	0.95395 (14)	0.0629 (5)	
H14	0.3067	1.3238	0.9047	0.076*	
C15	0.33854 (13)	1.15925 (15)	0.92883 (13)	0.0593 (5)	
H15	0.3181	1.1414	0.8623	0.071*	
C16	0.38761 (16)	1.44487 (18)	1.16989 (16)	0.0882 (7)	
H16A	0.3474	1.4126	1.1956	0.132*	
H16B	0.3846	1.5257	1.1720	0.132*	
H16C	0.4534	1.4205	1.2096	0.132*	
C17	0.07336 (13)	0.77001 (16)	0.11542 (13)	0.0607 (5)	
C18	0.11848 (11)	0.95659 (14)	0.08575 (11)	0.0473 (4)	
C19	0.14586 (11)	0.91583 (14)	0.02099 (12)	0.0507 (4)	
H19	0.1691	0.9653	−0.0105	0.061*	
C20	0.16909 (13)	0.75191 (15)	−0.06827 (14)	0.0608 (5)	
H20	0.1914	0.7993	−0.1020	0.073*	
C21	0.16469 (14)	0.63793 (17)	−0.08482 (14)	0.0690 (5)	
H21	0.1846	0.6087	−0.1292	0.083*	
C22	0.13085 (14)	0.56579 (17)	−0.03610 (14)	0.0713 (6)	
H22	0.1281	0.4884	−0.0476	0.086*	
C23	0.10135 (13)	0.60905 (16)	0.02928 (14)	0.0664 (5)	
H23	0.0785	0.5609	0.0620	0.080*	
C24	0.10552 (12)	0.72472 (15)	0.04675 (12)	0.0510 (4)	
C25	0.14046 (11)	0.79797 (14)	−0.00148 (12)	0.0477 (4)	
C26	0.12307 (11)	1.07354 (14)	0.11878 (11)	0.0471 (4)	
C27	0.09425 (12)	1.10360 (15)	0.18924 (12)	0.0575 (5)	
H27	0.0723	1.0476	0.2167	0.069*	
C28	0.09700 (13)	1.21374 (15)	0.22010 (13)	0.0584 (5)	
H28	0.0772	1.2312	0.2675	0.070*	
C29	0.12929 (12)	1.29732 (15)	0.18019 (13)	0.0517 (5)	
C30	0.15888 (13)	1.26986 (15)	0.10993 (14)	0.0621 (5)	
H30	0.1811	1.3261	0.0829	0.074*	
C31	0.15544 (13)	1.16016 (15)	0.08009 (13)	0.0595 (5)	
H31	0.1753	1.1432	0.0326	0.071*	
C32	0.09712 (14)	1.44297 (16)	0.26951 (14)	0.0770 (6)	
H32A	0.0317	1.4163	0.2434	0.115*	
H32B	0.0979	1.5238	0.2741	0.115*	
H32C	0.1358	1.4114	0.3345	0.115*	

### Atomic displacement parameters (Å^2^)

**Table:**

	*U*^11^	*U*^22^	*U*^33^	*U*^12^	*U*^13^	*U*^23^
O1	0.1390 (14)	0.0681 (9)	0.0507 (8)	0.0230 (9)	0.0355 (9)	0.0148 (7)
O2	0.0798 (9)	0.0542 (8)	0.0436 (7)	0.0102 (6)	0.0262 (6)	0.0041 (6)
O3	0.0977 (11)	0.0546 (9)	0.0764 (10)	0.0029 (7)	0.0407 (8)	−0.0108 (7)
O4	0.1466 (14)	0.0660 (9)	0.1082 (11)	−0.0176 (9)	0.0991 (11)	−0.0002 (8)
O5	0.0785 (9)	0.0537 (8)	0.0633 (8)	−0.0091 (6)	0.0473 (7)	−0.0027 (6)
O6	0.0808 (9)	0.0559 (9)	0.0723 (9)	−0.0028 (7)	0.0431 (7)	−0.0091 (7)
C1	0.0737 (14)	0.0577 (13)	0.0510 (12)	0.0102 (10)	0.0295 (11)	0.0063 (10)
C2	0.0468 (10)	0.0532 (12)	0.0422 (10)	0.0010 (8)	0.0216 (8)	0.0069 (9)
C3	0.0550 (11)	0.0513 (12)	0.0421 (10)	0.0013 (8)	0.0222 (9)	0.0070 (8)
C4	0.0612 (13)	0.0649 (14)	0.0481 (11)	−0.0035 (10)	0.0207 (10)	−0.0031 (9)
C5	0.0642 (13)	0.0718 (15)	0.0568 (13)	−0.0066 (11)	0.0220 (11)	−0.0176 (11)
C6	0.0734 (14)	0.0573 (13)	0.0729 (14)	0.0018 (10)	0.0313 (12)	−0.0088 (11)
C7	0.0747 (14)	0.0571 (14)	0.0642 (13)	0.0108 (10)	0.0343 (11)	0.0048 (10)
C8	0.0505 (11)	0.0533 (12)	0.0491 (11)	0.0024 (9)	0.0249 (9)	0.0002 (9)
C9	0.0424 (10)	0.0560 (12)	0.0446 (11)	−0.0041 (8)	0.0208 (9)	−0.0018 (9)
C10	0.0425 (10)	0.0514 (11)	0.0458 (10)	−0.0009 (8)	0.0203 (8)	0.0010 (9)
C11	0.0613 (12)	0.0582 (13)	0.0469 (11)	0.0034 (9)	0.0261 (9)	0.0023 (9)
C12	0.0663 (13)	0.0611 (13)	0.0477 (11)	0.0006 (10)	0.0271 (10)	−0.0052 (10)
C13	0.0554 (12)	0.0506 (13)	0.0622 (13)	−0.0029 (9)	0.0296 (10)	−0.0074 (10)
C14	0.0723 (14)	0.0546 (13)	0.0527 (12)	0.0058 (10)	0.0215 (10)	0.0072 (10)
C15	0.0704 (13)	0.0562 (13)	0.0458 (11)	0.0014 (10)	0.0226 (10)	−0.0015 (9)
C16	0.1071 (18)	0.0744 (16)	0.0891 (16)	−0.0108 (13)	0.0508 (14)	−0.0317 (13)
C17	0.0746 (14)	0.0555 (13)	0.0621 (12)	−0.0061 (10)	0.0404 (11)	0.0017 (10)
C18	0.0462 (10)	0.0534 (12)	0.0451 (10)	−0.0041 (8)	0.0236 (9)	0.0041 (8)
C19	0.0535 (11)	0.0532 (12)	0.0514 (10)	−0.0030 (8)	0.0296 (9)	0.0036 (9)
C20	0.0659 (13)	0.0609 (14)	0.0676 (12)	−0.0032 (10)	0.0413 (11)	−0.0041 (10)
C21	0.0744 (14)	0.0692 (14)	0.0755 (14)	0.0024 (11)	0.0451 (12)	−0.0095 (11)
C22	0.0824 (15)	0.0551 (13)	0.0783 (14)	0.0029 (10)	0.0391 (12)	−0.0053 (11)
C23	0.0775 (14)	0.0581 (14)	0.0695 (13)	−0.0065 (10)	0.0393 (11)	−0.0005 (10)
C24	0.0512 (11)	0.0520 (12)	0.0501 (10)	−0.0006 (9)	0.0237 (9)	−0.0003 (9)
C25	0.0428 (10)	0.0552 (12)	0.0444 (10)	0.0001 (8)	0.0198 (9)	0.0005 (9)
C26	0.0442 (10)	0.0524 (11)	0.0453 (10)	−0.0005 (8)	0.0213 (8)	0.0017 (8)
C27	0.0646 (12)	0.0618 (13)	0.0562 (11)	−0.0057 (9)	0.0365 (10)	0.0009 (9)
C28	0.0684 (13)	0.0622 (13)	0.0551 (11)	−0.0019 (10)	0.0377 (10)	−0.0064 (10)
C29	0.0519 (11)	0.0509 (12)	0.0523 (11)	0.0009 (9)	0.0243 (9)	−0.0022 (9)
C30	0.0728 (14)	0.0543 (12)	0.0749 (13)	−0.0098 (10)	0.0478 (12)	−0.0024 (10)
C31	0.0731 (13)	0.0595 (13)	0.0634 (12)	−0.0060 (10)	0.0465 (11)	−0.0055 (10)
C32	0.0862 (15)	0.0708 (15)	0.0819 (14)	0.0036 (11)	0.0459 (13)	−0.0175 (11)

### Geometric parameters (Å, °)

**Table:**

O1—C1	1.2048 (19)	C14—H14	0.9300
O2—C1	1.381 (2)	C15—H15	0.9300
O2—C2	1.3842 (18)	C16—H16A	0.9600
O3—C13	1.3672 (19)	C16—H16B	0.9600
O3—C16	1.422 (2)	C16—H16C	0.9600
O4—C17	1.2032 (19)	C17—C24	1.454 (2)
O5—C17	1.3765 (19)	C18—C19	1.332 (2)
O5—C18	1.3794 (17)	C18—C26	1.464 (2)
O6—C29	1.3700 (18)	C19—C25	1.431 (2)
O6—C32	1.4272 (19)	C19—H19	0.9300
C1—C8	1.453 (2)	C20—C21	1.369 (2)
C2—C3	1.330 (2)	C20—C25	1.396 (2)
C2—C10	1.465 (2)	C20—H20	0.9300
C3—C9	1.431 (2)	C21—C22	1.386 (3)
C3—H3	0.9300	C21—H21	0.9300
C4—C5	1.373 (2)	C22—C23	1.373 (2)
C4—C9	1.398 (2)	C22—H22	0.9300
C4—H4	0.9300	C23—C24	1.391 (2)
C5—C6	1.377 (2)	C23—H23	0.9300
C5—H5	0.9300	C24—C25	1.400 (2)
C6—C7	1.367 (2)	C26—C27	1.387 (2)
C6—H6	0.9300	C26—C31	1.389 (2)
C7—C8	1.394 (2)	C27—C28	1.380 (2)
C7—H7	0.9300	C27—H27	0.9300
C8—C9	1.394 (2)	C28—C29	1.373 (2)
C10—C11	1.387 (2)	C28—H28	0.9300
C10—C15	1.394 (2)	C29—C30	1.384 (2)
C11—C12	1.375 (2)	C30—C31	1.369 (2)
C11—H11	0.9300	C30—H30	0.9300
C12—C13	1.369 (2)	C31—H31	0.9300
C12—H12	0.9300	C32—H32A	0.9600
C13—C14	1.391 (2)	C32—H32B	0.9600
C14—C15	1.369 (2)	C32—H32C	0.9600
			
C1—O2—C2	122.83 (14)	H16A—C16—H16C	109.5
C13—O3—C16	117.94 (15)	H16B—C16—H16C	109.5
C17—O5—C18	123.25 (13)	O4—C17—O5	116.63 (16)
C29—O6—C32	117.23 (14)	O4—C17—C24	126.34 (18)
O1—C1—O2	116.16 (16)	O5—C17—C24	117.03 (15)
O1—C1—C8	126.72 (19)	C19—C18—O5	119.92 (15)
O2—C1—C8	117.12 (16)	C19—C18—C26	127.93 (15)
C3—C2—O2	120.00 (16)	O5—C18—C26	112.14 (13)
C3—C2—C10	128.46 (16)	C18—C19—C25	121.77 (15)
O2—C2—C10	111.54 (14)	C18—C19—H19	119.1
C2—C3—C9	121.76 (16)	C25—C19—H19	119.1
C2—C3—H3	119.1	C21—C20—C25	120.88 (17)
C9—C3—H3	119.1	C21—C20—H20	119.6
C5—C4—C9	120.42 (17)	C25—C20—H20	119.6
C5—C4—H4	119.8	C20—C21—C22	120.60 (18)
C9—C4—H4	119.8	C20—C21—H21	119.7
C4—C5—C6	120.79 (18)	C22—C21—H21	119.7
C4—C5—H5	119.6	C23—C22—C21	119.68 (19)
C6—C5—H5	119.6	C23—C22—H22	120.2
C7—C6—C5	120.17 (19)	C21—C22—H22	120.2
C7—C6—H6	119.9	C22—C23—C24	120.24 (18)
C5—C6—H6	119.9	C22—C23—H23	119.9
C6—C7—C8	119.70 (18)	C24—C23—H23	119.9
C6—C7—H7	120.2	C23—C24—C25	120.39 (16)
C8—C7—H7	120.2	C23—C24—C17	119.93 (16)
C9—C8—C7	120.83 (16)	C25—C24—C17	119.69 (17)
C9—C8—C1	119.83 (17)	C20—C25—C24	118.20 (16)
C7—C8—C1	119.34 (17)	C20—C25—C19	123.49 (15)
C8—C9—C4	118.06 (16)	C24—C25—C19	118.31 (15)
C8—C9—C3	118.44 (15)	C27—C26—C31	116.70 (16)
C4—C9—C3	123.50 (16)	C27—C26—C18	121.72 (15)
C11—C10—C15	116.53 (16)	C31—C26—C18	121.58 (14)
C11—C10—C2	122.33 (16)	C28—C27—C26	122.33 (16)
C15—C10—C2	121.13 (15)	C28—C27—H27	118.8
C12—C11—C10	122.34 (17)	C26—C27—H27	118.8
C12—C11—H11	118.8	C29—C28—C27	119.43 (16)
C10—C11—H11	118.8	C29—C28—H28	120.3
C13—C12—C11	119.88 (17)	C27—C28—H28	120.3
C13—C12—H12	120.1	O6—C29—C28	124.97 (16)
C11—C12—H12	120.1	O6—C29—C30	115.46 (16)
O3—C13—C12	125.53 (17)	C28—C29—C30	119.57 (17)
O3—C13—C14	115.03 (17)	C31—C30—C29	120.16 (17)
C12—C13—C14	119.44 (17)	C31—C30—H30	119.9
C15—C14—C13	119.93 (17)	C29—C30—H30	119.9
C15—C14—H14	120.0	C30—C31—C26	121.81 (16)
C13—C14—H14	120.0	C30—C31—H31	119.1
C14—C15—C10	121.87 (16)	C26—C31—H31	119.1
C14—C15—H15	119.1	O6—C32—H32A	109.5
C10—C15—H15	119.1	O6—C32—H32B	109.5
O3—C16—H16A	109.5	H32A—C32—H32B	109.5
O3—C16—H16B	109.5	O6—C32—H32C	109.5
H16A—C16—H16B	109.5	H32A—C32—H32C	109.5
O3—C16—H16C	109.5	H32B—C32—H32C	109.5
			
C2—O2—C1—O1	−179.82 (16)	C18—O5—C17—O4	−179.43 (17)
C2—O2—C1—C8	−0.1 (2)	C18—O5—C17—C24	0.4 (2)
C1—O2—C2—C3	−0.7 (2)	C17—O5—C18—C19	1.2 (2)
C1—O2—C2—C10	179.49 (14)	C17—O5—C18—C26	−177.86 (14)
O2—C2—C3—C9	0.4 (2)	O5—C18—C19—C25	−1.3 (2)
C10—C2—C3—C9	−179.86 (14)	C26—C18—C19—C25	177.58 (15)
C9—C4—C5—C6	0.8 (3)	C25—C20—C21—C22	0.6 (3)
C4—C5—C6—C7	−1.1 (3)	C20—C21—C22—C23	0.1 (3)
C5—C6—C7—C8	0.0 (3)	C21—C22—C23—C24	−0.1 (3)
C6—C7—C8—C9	1.4 (3)	C22—C23—C24—C25	−0.5 (3)
C6—C7—C8—C1	−178.60 (17)	C22—C23—C24—C17	179.46 (17)
O1—C1—C8—C9	−179.05 (18)	O4—C17—C24—C23	−2.0 (3)
O2—C1—C8—C9	1.3 (2)	O5—C17—C24—C23	178.20 (15)
O1—C1—C8—C7	1.0 (3)	O4—C17—C24—C25	177.94 (19)
O2—C1—C8—C7	−178.74 (15)	O5—C17—C24—C25	−1.9 (2)
C7—C8—C9—C4	−1.7 (2)	C21—C20—C25—C24	−1.1 (3)
C1—C8—C9—C4	178.31 (15)	C21—C20—C25—C19	178.23 (16)
C7—C8—C9—C3	178.43 (14)	C23—C24—C25—C20	1.1 (2)
C1—C8—C9—C3	−1.6 (2)	C17—C24—C25—C20	−178.86 (15)
C5—C4—C9—C8	0.6 (2)	C23—C24—C25—C19	−178.30 (15)
C5—C4—C9—C3	−179.54 (16)	C17—C24—C25—C19	1.8 (2)
C2—C3—C9—C8	0.7 (2)	C18—C19—C25—C20	−179.51 (16)
C2—C3—C9—C4	−179.12 (16)	C18—C19—C25—C24	−0.2 (2)
C3—C2—C10—C11	179.61 (16)	C19—C18—C26—C27	−178.18 (17)
O2—C2—C10—C11	−0.6 (2)	O5—C18—C26—C27	0.8 (2)
C3—C2—C10—C15	−0.3 (3)	C19—C18—C26—C31	2.6 (3)
O2—C2—C10—C15	179.50 (14)	O5—C18—C26—C31	−178.40 (15)
C15—C10—C11—C12	−0.2 (2)	C31—C26—C27—C28	0.1 (3)
C2—C10—C11—C12	179.95 (15)	C18—C26—C27—C28	−179.13 (15)
C10—C11—C12—C13	0.7 (3)	C26—C27—C28—C29	0.0 (3)
C16—O3—C13—C12	2.0 (3)	C32—O6—C29—C28	−6.6 (2)
C16—O3—C13—C14	−178.23 (17)	C32—O6—C29—C30	173.92 (16)
C11—C12—C13—O3	179.38 (15)	C27—C28—C29—O6	−179.69 (15)
C11—C12—C13—C14	−0.4 (3)	C27—C28—C29—C30	−0.2 (3)
O3—C13—C14—C15	179.79 (16)	O6—C29—C30—C31	179.88 (15)
C12—C13—C14—C15	−0.4 (3)	C28—C29—C30—C31	0.4 (3)
C13—C14—C15—C10	0.9 (3)	C29—C30—C31—C26	−0.3 (3)
C11—C10—C15—C14	−0.7 (3)	C27—C26—C31—C30	0.0 (3)
C2—C10—C15—C14	179.22 (15)	C18—C26—C31—C30	179.28 (16)

## References

[bb1] Abid, O.-U.-R., Qadeer, G., Rama, N. H., Ruzicka, A. & Padelkova, Z. (2008). *Acta Cryst.* E**64**, o2018. 10.1107/S1600536808030274 PMC2959484 21201213

[bb2] Abid, O., Rama, N. H., Qadeer, G., Khan, G. S. & Lu, X.-M. (2006). *Acta Cryst.* E**62**, o2895–o2896.

[bb3] Barry, R. D. (1964). *Chem. Rev.* **64**, 229–260.

[bb4] Bruker (2004). *SMART* and *SAINT* Bruker AXS Inc., Madison, Wisconsin, USA.

[bb5] Canendo, L. M., Puents, J. L. F. & Baz, J. P. (1997). *J. Antibiot.* **50**, 175–176.

[bb6] Farrugia, L. J. (1997). *J. Appl. Cryst.* **30**, 565.

[bb7] Hathwar, V. R., Manivel, P., Nawaz Khan, F. & Guru Row, T. N. (2007). *Acta Cryst.* E**63**, o3707.

[bb8] Hill, R. A. (1986). *Fortschr. Chem. Org. Naturst.* **49**, 1–78.

[bb9] Sheldrick, G. M. (1996). *SADABS* University of Göttingen, Germany.

[bb10] Sheldrick, G. M. (2008). *Acta Cryst.* A**64**, 112–122. 10.1107/S0108767307043930 18156677

[bb15] Spek, A. L. (2003). *J. Appl. Cryst.* **36**, 7–13.

[bb12] Watkin, D. J., Pearce, L. & Prout, C. K. (1993). *CAMERON* Chemical Crystallography Laboratory, University of Oxford, England.

[bb13] Whyte, A. C., Gloer, J. B., Scott, J. A. & Malloch, D. (1996). *J. Nat. Prod.* **59**, 765–769. 10.1021/np9603232 8792624

